# The Impact of Allogeneic Hematopoietic Stem Cell Transplantation on Kidney Function in Children—A Single Center Experience

**DOI:** 10.3390/jcm10051113

**Published:** 2021-03-07

**Authors:** Kinga Musiał, Krzysztof Kałwak, Danuta Zwolińska

**Affiliations:** 1 Department of Pediatric Nephrology, Wrocław Medical University, 50-556 Wrocław, Poland; danuta.zwolinska@umed.wroc.pl; 2 Department of Bone Marrow Transplantation, Oncology and Pediatric Hematology, Wrocław Medical University, 50-556 Wrocław, Poland; krzysztof.kalwak@umed.wroc.pl

**Keywords:** acute kidney injury, estimated glomerular filtration rate, hyperfiltration, pRIFLE criteria

## Abstract

Background: Knowledge about the impact of allogeneic hematopoietic stem cell transplantation (alloHSCT) on renal function in children is still limited. Objectives: The aim of the study was to evaluate kidney function in children undergoing alloHSCT, with special focus on differences between patients transplanted due to oncological and non-oncological indications. Materials and Methods: The data of 135 children undergoing alloHSCT were analyzed retrospectively. The serum creatinine and estimated glomerular filtration rate (eGFR) values were estimated before transplantation at 24 h; 1, 2, 3, 4 and 8 weeks; and 3 and 6 months after alloHSCT. Then, acute kidney injury (AKI) incidence was assessed. Results: Oncological children presented with higher eGFR values and more frequent hyperfiltration rates than non-oncological children before alloHSCT and until the 4th week after transplantation. The eGFR levels rose significantly after alloHSCT, returned to pre-transplant records after 2–3 weeks, and decreased gradually until the 6th month. AKI incidence was comparable in oncological and non-oncological patients. Conclusions: Children undergoing alloHSCT due to oncological and non-oncological reasons demonstrate the same risk of AKI, but oncological patients may be more prone to sustained renal injury. Serum creatinine and eGFR seem to be insufficient tools to assess kidney function in the early post-alloHSCT period, when hyperfiltration prevails, yet they reveal significant differences in long-term observation.

## 1. Introduction

The indications for hematopoietic stem cell transplantation (HSCT) in children are expanding constantly [1,2,3,4]. Apart from dominating oncological reasons for this procedure, there are a growing number of other diseases efficiently treated with HSCT such as aplastic anemia, immunodeficiencies and inborn metabolic disorders [1,3]. Aggressive therapy, including myeloablative conditioning or prophylaxis against graft versus host disease, is responsible for serious side effects and life-threatening complications [5,6,7,8]. The abovementioned conditions, together with nephrotoxicity related to previous chemotherapy, hypertension or infections, aggravate the risk of developing acute kidney injury (AKI) [9]. Moreover, up to one third of children with post-HSCT AKI may develop chronic kidney disease [10,11]. However, few publications estimating the scale of this phenomenon exist [12,13].

The comparison of data from national reports suggests that AKI occurrence strongly depends on the method of its assessment, and pRIFLE (pediatric Risk, Injury, Failure, Loss and End stage kidney disease) criteria reveal higher AKI incidence than the KDIGO (Kidney Disease Improving Global Outcomes) definition [14,15,16]. The existence of such discrepancies triggers the search for new markers of early kidney injury in this population [17]. Our previous study in children undergoing allogeneic hematopoietic stem cell transplantation (alloHSCT) concerned the assessment of classical markers of kidney function (serum creatinine, cystatin C) in confrontation with known (urinary kidney injury molecule (KIM)-1) and new (urinary clusterin) damage markers [18]. The urinary concentrations of examined indices showed elevation already before alloHSCT when serum creatinine was normal. Moreover, this elevation of damage markers continued, or was even aggravated, just after transplantation, and persisted until the 2nd–4th week [18].

These results inspired us to analyze retrospectively, with the use of classical indices, the kidney function of children undergoing alloHSCT, with special focus on the specificity of pediatric procedures during preparation for HSCT and after the transplantation, and potential differences between the subpopulations of patients treated due to oncological and non-oncological reasons.

## 2. Aim of Study

The objective of the study was to assess kidney function based on the serum creatinine and estimated glomerular filtration rate (eGFR) values in children undergoing alloHSCT due to oncological and non-oncological reasons in the early, intermediate, and late post-transplantation periods. The second goal was to analyze whether the variability of serum creatinine and eGFR values in the course of alloHSCT, as well as the choice of criteria defining stages of renal injury, may modify AKI incidence in the pediatric population.

## 3. Materials and Methods

The evaluation of kidney function relied on hematological protocols assessing serum creatinine at fixed time points. Serum creatinine concentration was measured by a modified Jaffe method before conditioning, at 24 h, 1, 2, 3, 4, 8 weeks, and 3 and 6 months after alloHSCT. eGFR was calculated for all time points based on the Schwarz formula [19]. The eGFR changes were confronted with the pre-transplantation values.

AKI was diagnosed based on the KDIGO definition, i.e., evaluating serum creatinine rise, and pRIFLE criteria assessing the degree of eGFR decrease [16]. The urine output criteria were not taken into account due to the lack of available data. Additionally, the children with a statistically significant eGFR decrease not exceeding 25% were included and classified as the patients at ”grade zero” [20]. Hyperfiltration was defined as eGFR ≥ 140 mL/min/1.73 m^2^, based on data from meta-analysis and recent pediatric experience [21,22]. 

### Statistical Analysis

Continuous variables were expressed as mean ± standard deviation (SD) and categorical variables as frequencies and percentages. The comparisons of continuous variables were performed with ANOVA and the Student’s *t*-test. The relations between categorical variables were tested with a chi-square test or Fisher’s exact test. General linear models with the results of a Wilks (W) test were used to describe the impact of group (oncological vs. non-oncological), previous chemotherapy or current therapeutic protocols on the serum creatinine and eGFR values. *p* value < 0.05 was considered significant. All calculations were carried out with the use of Statistica v.13.3 (StatSoft Inc.,Tulsa, OK, USA).

## 4. Results

### 4.1. Patient Characteristics

The study focused on a retrospective analysis (years 2016–2018) of the medical records of 173 children undergoing first alloHSCT and 5 children undergoing next alloHSCT in the Department of Bone Marrow Transplantation, Pediatric Oncology and Haematology. The observation started before introducing conditioning therapy and then continued through the early (after 24 h and after 1, 2, 3 and 4 weeks), intermediate (after 8 weeks and after 3 months) and late (after 6 months) post-transplantation periods.

The exclusion criteria for the patients were as follows: age below 2 years (due to disproportionately low physiological eGFR in comparison to older children) and over 18 years. Out of 178 children, 78 boys and 57 girls met the age criteria (mean age 8.27 ± 5.14 years). They were divided into two groups depending on the indication for allotransplantation. The first group, containing 89 patients, was qualified for transplantation due to oncological reasons (acute leukemias in 84% of cases). The second group consisted of 46 patients who underwent alloHSCT following non-oncological indications (severe aplastic anemia in 48% of cases). Detailed demographic and clinical data are shown in Table 1.

Conditioning therapy concerned myeloablative (busulfan, cyclophosphamide, fludarabine, or fludarabine, treosulfan, thiotepa) or non-myeloablative (cyclophosphamide, fludarabine) regimens. In the majority of patients, the graft versus host disease (GvHD) prophylaxis protocol contained pre-HSCT antithymocyte globulin (ATG), cyclosporine A since 1 day before HSCT and methotrexate given on the 1st, 3rd and 6th day after transplantation. In total, 98 out of 135 patients developed GvHD (Table 1). 

Patients transplanted due to oncological reasons were nearly 2-fold more numerous than children undergoing alloHSCT because of non-oncological indications. The evaluation of kidney function in both groups by means of serum creatinine and glomerular filtration rate revealed several discrepancies.

### 4.2. Serum Creatinine and eGFR Values

Serum creatinine values before alloHSCT were normal and comparable in both groups (Table 2). They decreased significantly the day after transplantation, and this diminishment persisted until the first (non-oncological patients) or second week (oncological patients) after alloHSCT (Table 2). In the meantime, the mean creatinine values in oncological children remained lower than those in non-oncological children. Serum creatinine in non-oncological children returned to pre-transplantation records after 2 weeks and remained stable until the 6th month, when they became significantly higher again. Oncological patients demonstrated the normalization of serum creatinine values after 3 weeks, yet the values started increasing again from the 4th week and did so until the 6th month after alloHSCT.

General linear models revealed the effect of group (W = 0.84; *p* = 0.02) and previous chemotherapy (W = 0.79; *p* = 0.02) on serum creatinine values after HSCT and 1, 2 and 4 weeks after HSCT, whereas the effect of cyclophosphamide treatment (W = 0.83; *p* = 0.007) was significant after HSCT and 2 and 4 weeks after HSCT.

The eGFR values before transplantation were, for the vast majority, above 90 mL/min/1.73 m^2^ in both groups, and none of the patients presented with eGFR < 60 mL/min/1.73 m^2^ (Table 1). The patients with eGFR not exceeding 140 mL/min/1.73 m^2^ were more numerous among non-oncological children, whereas the number of patients with hyperfiltration was significantly higher in the oncological group when compared to non-oncological children (Table 1). The highest eGFR values (hyperfiltration in 72% of oncological children and in 50% of non-oncological patients) were observed in both groups 1 day and 1 week after alloHSCT (Table 2). The eGFR values returned to the records observed before the treatment after 2 weeks in non-oncological children and after 3 weeks in oncological patients (Table 2). Subsequently, the mean eGFR values in oncological children remained lower than before alloHSCT and continued decreasing from the 4th week until the 6th month of observation (Table 2). The mean eGFR values in non-oncological patients became comparable to the pre-transplantation records 3 weeks after alloHSCT and remained as such until the 6th month of observation (Table 2). 

Meanwhile, the mean eGFR values in oncological patients were significantly higher than those in non-oncological ones already before alloHSCT and until the 4th week after transplantation (Table 2). After 6 months, the discrepancy tended to reach statistical significance in favor of higher values in non-oncological patients (*p* = 0.058).

General linear models revealed the effect of the group (W = 0.76; *p* = 0.005) on eGFR values both before and after alloHSCT until the 4th week of observation. The impact of previous chemotherapy (W = 0.79; *p* = 0.02) was significant after HSCT and 2 weeks after HSCT, whereas the effect of cyclophosphamide treatment (W = 0.83; *p* = 0.007) was significant only 2 weeks after HSCT.

### 4.3. The incidence of AKI

The comparison of AKI incidence in both subgroups showed comparable results (Table 3). Fifty-four percent of patients demonstrated features of kidney damage according to the pRIFLE criteria during the entire time of observation. The risk stage (grade 1) was noticed at least once in 58 patients and the injury stage (grade 2) in 14 patients. None of the patients experienced an eGFR decrease of 75% (grade 3). The most numerous group (with a preponderance of oncological patients) demonstrated a statistically significant eGFR decrease not exceeding 25% (grade 0) (Table 3).

When KDIGO guidelines were taken into account, only 26% of children fulfilled the criteria of AKI. AKI stage 1 concerned 27 patients, whereas stage 2 was diagnosed in 9 children.

Nearly 13% of the patients discontinued the follow-up after 3 months because of the transfer to home hematological centers. Among the remaining population, 78.8% of oncological patients demonstrated significantly diminished eGFR values compared to pre-transplantation ones. In the case of non-oncological patients, such decrease did not reach statistical significance. In total, 8.8% of children had eGFR values between 60 and 89 mL/min/1.73 m^2^, while in just 1.5%, glomerular filtration dropped below 60 mL/min/1.73 m^2^. Hyperfiltration was still present in 34% of oncological and 20% of non-oncological patients. 

Ten patients (0.7%) died during the observation period. Nine deaths were caused by late non-nephrological complications, and only one case (20 days after alloHSCT) showed a direct connection with the procedure of allotransplantation. Nobody required renal replacement therapy.

## 5. Discussion

Our study has shown the challenges in assessment of renal function and discrepant results of kidney injury analysis in the population of children undergoing alloHSCT.

Although the post-HSCT AKI has undergone in-depth analyses in adults, it still affects more than 50% of patients undergoing transplantation [23,24]. Similar evaluations in the pediatric population have concentrated on long-term complications and mortality rates rather than features of early kidney injury [12,13,14,15]. The shortage of data analyzing renal function in children after HSCT, specifically in the early post-transplantation period, may result from several difficulties that such evaluation imposes.

Diagnostic discrepancies are the first challenge in the evaluation of pediatric kidney function. For years, there have been various criteria for AKI diagnoses, adjusted mainly to adults and taking into account absolute serum creatinine values. From that point of view, pRIFLE criteria seemed a better option for children, since they took into account the eGFR decrease [16]. However, no method seems tailored for oncological patients, in whom multiple therapy-related factors such as weight loss, catabolism, inflammation or forced diuresis may influence serum creatinine or cystatin C, used to assess eGFR [25]. Moreover, various equations calculating eGFR in this population may give discrepant results [26]. 

The routine procedures performed during conditioning and up to 3 weeks after alloHSCT can make the interpretation of the results even more difficult. First, patients in the early phase (up to 3 weeks) are nourished and given additional fluids intravenously at the amount of 3 L/m^2^/day, with subsequent administration of diuretics if needed. Thus, altered fluid and calorie balances could modify the value of eGFR and make its interpretation a challenge. Even though the AWARE (Assessment of Worldwide Acute Kidney Injury, Renal Angina and Epidemiology in Critically Ill Children) study has suggested a promising perspective, taking into account serum creatinine changes in relation to overhydration and diuresis, the restrictive fluid balance control in this population is not everyday practice outside intensive care units [27]. 

All abovementioned discrepancies were seen among our patients. Before treatment, all of them demonstrated normal serum creatinine concentration, diminishing just after HSCT and returning to pre-transplantation values 2–3 weeks later. The reasons for such decrement could be multiple, including overhydration, catabolism and weight loss. Our analysis suggests that serum creatinine values in the early post-HSCT period also depend on previous chemotherapy and cyclophosphamide use. Irrespective of the background, in practice, any potential sign of kidney injury during this early period would have been masked if serum creatinine had been the only marker evaluated. The hypothesis on possible renal injury in this early period was partly confirmed by our preliminary study assessing various indices in the early post-transplantation period [18]. 

The observation time of this study, extending beyond the first month after transplantation, revealed an increasing tendency in serum creatinine values. Oncological patients dealt with it from the 4th week until the 6th month after alloHSCT, whereas in non-oncological children, a significant elevation was revealed no sooner than 6 months after transplantation. Undoubtedly, this could be the sign of a potential risk of sustained renal dysfunction and should be verified in the course of future research.

Therefore, we assumed that the concomitant assessment of eGFR values could be of added value. Indeed, a tendency toward hyperfiltration, more evident in the case of oncological children, was seen even before conditioning therapy. This observation was concordant with reports on high incidence of hyperfiltration in children with malignancies [28]. The authors proved its connection to a hypermetabolic state and a tendency toward increasing eGFR values with subsequent cycles of chemotherapy [28]. The positive correlation between increased eGFR values and intensity of immunosuppression suggested that hyperfiltration could be a surrogate marker of renal dysfunction. Another recent study has confirmed the role of hyperfiltration as a marker of incipient renal dysfunction in the pediatric population of cancer survivors [29]. In our population, the significantly elevated eGFR values in oncological patients compared to non-oncological ones seemed to confirm this assumption. The preceding chemotherapy, concerning all oncological children and only one third of non-oncological children, was assumed to be a possible trigger factor for tubular damage and increased diuresis.

The nephrotoxic burden due to the conditioning therapy also had to be taken into account in the overall analysis of factors triggering kidney damage. Nearly everybody obtained cyclosporin. Treosulfan was used more often in oncological patients, whereas cyclophosphamide was applied more often in non-oncological children. Both of them are excreted by the kidneys. Extensive hydration with subsequent diuretics are routine procedures after HSCT and they seem effective in the prevention of potential nephrotoxic side effects. However, the precise evaluation of the impact of a single nephrotoxic agent in the background of their multiple use at the same time remained a challenge. Our analysis has revealed the significant effect of previous chemotherapy and, in regard to current treatment, cyclophosphamide impact on the kidney function, but only until the 4th week after alloHSCT.

Another surprising finding concerned children in whom the decrease in eGFR values, although statistically significant, did not reach 25%. They were the most numerous group among all patients, demonstrating eGFR decrease throughout the observation period. Moreover, their number increased during follow up and crossed the border of 40% after 6 months, both in oncological and non-oncological patients. It could be hypothesized that this persistent eGFR decrease, similar for both groups, is a direct consequence of HSCT-related therapy and complications. Indeed, except for treosulfan, thiotepa and ATG, there were no major differences in therapy between oncological and non-oncological patients. Likewise, these groups did not differ regarding the frequency or severity of complications. Therefore, the treatment protocols characteristic of alloHSCT are potentially suspected of triggering or aggravating chronic kidney dysfunction, irrespective of the previous medical history. However, a longer time of follow-up after alloHSCT is required to confirm this hypothesis. 

On the other hand, the results of our observation have raised several doubts about the mode of interpretation of AKI incidence, especially based on the pRIFLE criteria, in this particular group of patients. After 6 months of observation, the eGFR values, although reduced by up to 50%, remained above the borderline value of 90 mL/min/1.73 m^2^ in 95% of patients. Moreover, many of them still demonstrated hyperfiltration. Having said that, is it proper to diagnose AKI in someone whose eGFR value remains within normal range, irrespective of the >25% reduction? If we assume that hyperfiltration in these patients was the consequence of chronic tubular damage prior to alloHSCT, would the subsequent decrease in eGFR imply functional overlap with the deterioration of glomerular function? Could this functional overlap be another example of tubuloglomerular feedback triggering hyperfiltration, described already in the case of high protein intake or diabetes [30,31]? The abovementioned doubts clearly show that assessing the spectrum of kidney injury in these patients only by the means of serum creatinine and eGFR narrows the perspective and limits the understanding of underlying processes responsible for renal dysfunction in the course of alloHSCT.

Unexpectedly, despite the differences in parameters of renal function between oncological and non-oncological patients, the incidence of AKI did not show discrepancies in both subgroups. However, pRIFLE criteria identified more than half of children as having AKI, whereas KDIGO definition allowed this diagnosis in only one fourth of them. This method-related bias was concordant with the estimations performed by other authors and confirmed that relying on one diagnostic tool may give unreliable and misleading results [12,14,15].

Summarizing, the presented results give way to strong suggestion that an additional method of renal function assessment (especially in the early period after alloHSCT) is needed for this particular group of patients. Probably, the use of renal damage markers will become obligatory, but their role in pediatric HSCT is yet to be established [17,18]. 

We also have to acknowledge the limitations of our study. First, this was a retrospective report and the data were collected according to hematological protocols, not taking into account all nephrological aspects. Thus, the information about urine output is missing, as well as measurements of cystatin C or assessment of creatinine by the enzymatic method. We are also aware of the heterogeneity of examined groups, especially in the case of non-oncological patients. Another limitation is the fact that most of the patients were under regular control only throughout the first 3 months, and then some of them moved to other hospitals or did not appear regularly for control examinations. Thus, the analysis beyond the period of 6 months has turned out to be irrelevant because of patient flow. 

## 6. Conclusions

Children undergoing alloHSCT due to oncological and non-oncological reasons present with the same risk of AKI, as defined by KDIGO and pRIFLE, but oncological patients may be more prone to sustained renal injury. Serum creatinine and eGFR seem to be insufficient tools to assess kidney function in the early post-transplantation period yet reveal significant differences in long-term observation. New damage markers may become an additional tool to verify the grade of renal dysfunction during the first months after alloHSCT when hyperfiltration is a common finding, and longer follow-up studies are needed.

## Figures and Tables

**Table 1:** Basic demographic and clinical data concerning the studied groups.

Features Assessed	Oncological Indication for AlloHSCT	Non-Oncological Indication for AlloHSCT
Mean age (years) ± SD at alloHSCT	9.88 ± 4.33	9.24 ± 4.76 ^#^
Girls/boys (*n*; %)	36 (40.4%)/53 (59.6%)	21 (45.7%)/25 (54.3%)
Mean body weight (kg) ± SD at alloHSCT	33.8 ± 18.6	31.5 ± 16.7 ^#^
Mean height (cm) ± SD at alloHSCT	134.5 ± 27	128.7 ± 25.6 ^#^
Mean body surface area (m^2^) ± SD at alloHSCT	1.1 ± 0.4	1.0 ± 0.4 ^#^
Previous chemotherapy (*n*; %)	89 (100%)	15 (33%) *
**Baseline kidney function**		
eGFR < 90 mL/min/1.73 m^2^ (*n*; %)	9 (10.1%)	2 (4.3%)
eGFR 90–140 mL/min/1.73 m^2^ (*n*; %)	33 (37.1%)	34 (73.9%) *
eGFR > 140 mL/min/1.73 m^2^ (*n*; %)	45 (50.6%)	10 (21.7%) *
**Donor type** (*n*; %)		
Unrelated	64 (72%)	34 (73.9%)
Sibling	19 (21.3%)	9 (19.6%)
Haploidentical	5 (5.6%)	3 (6.5%)
**Matching** (*n*; %)		
10/10	55 (62%)	31 (67.4%)
<10/10	34 (38%)	15 (32.6%)
**Stem cell source** (*n*; %)		
Peripheral blood	77 (86.5%)	37 (80.4%)
Bone marrow	12 (13.5%)	9 (19.6%)
**Conditioning treatment**		
Treosulfan	60 (67.4%)	9 (19.6%) *
Busulfan	17 (19.1%)	15 (32.6%)
Fludarabine	76 (85.3%)	43 (93.5%)
Thiotepa	70 (78.7%)	16 (34.7%) *
Melphalan	8 (9%)	5 (10.9%)
Cyclophosphamide	6 (6.7%)	25 (54.3%) *
Etoposide	9 (10.1%)	0
Total body irradiation (TBI)	9 (10.1%)	0
**Graft versus host disease (GvHD) prophylaxis**		
Antithymocyte globulin (ATG)	64 (71.9%)	44 (95.7%) *
Cyclosporin	87 (97.8%)	45 (97.8%)
Methotrexate	66 (74.2%)	39 (84.8%)
Mycophenolate mofetil (MMF)	10 (11.2%)	10 (21.7%)
Steroids	6 (6.7%)	3 (6.5%)
**Graft versus host disease (GvHD) incidence**		
Acute GvHD	49 (55.1%)	28 (60.9%)
Chronic GvHD	13 (14.6%)	8 (17.4%)
**Viral infections**		
Adenovirus (ADV)	24 (27%)	14 (30.4%)
BK virus (BKV)	60 (67.4%)	26 (56.5%)
Cytomegalovirus (CMV)	34 (38.2%)	13 (28.3%)
Epstein-Barr virus (EBV)	23 (25.8%)	12 (26.1%)
**Bacterial infections**	18 (20.2%)	5 (10.9%)
**Fungal infections**	1 (1.1%)	1 (2.2%)

Allo-HSCT—allogeneic hematopoietic stem cell transplantation, SD—standard deviation, eGFR—estimated glomerular filtration rate * *p* < 0.05 in chi square Pearson test; ^#^ *p* > 0.3 in Student’s *t*-test.

**Table 2:** Mean serum creatinine and estimated glomerular filtration rate (eGFR) values in examined groups.

Parameters Assessed Mean ± SD	Patients O/NO	before AlloHSCT	1 Day after AlloHSCT	2 Weeks after AlloHSCT	4 Weeks after AlloHSCT	3 Months after AlloHSCT	6 Months after AlloHSCT
**Serum Creatinine (mg/dL)**	O	0.57 ± 0.20	0.49 ± 0.18 ^a b^	0.52 ± 0.21 ^b^	0.61 ± 0.19 ^b^	0.69 ± 0.26 ^b^	0.63 ± 0.18 ^b^
	NO	0.60 ± 0.17	0.53 ± 0.16 ^b^	0.57 ± 0.17	0.64 ± 0.19	0.67 ± 0.25	0.67 ± 0.20 ^b^
**eGFR (mL/min/1.73 m^2^)**	O	149 ± 48 ^a^	174 ± 58 ^a b^	168 ± 62 ^a b^	138 ± 48 ^a b^	124 ± 46 ^b^	131 ± 42 ^b^
	NO	125 ± 26	143 ± 28 ^b^	131 ± 28	120 ± 27	118 ± 30	117 ± 29

eGFR—estimated glomerular filtration rate; O—oncological patients; NO—non-oncological patients; alloHSCT—hematopoietic stem cell allotransplantation; **^a^** *p* < 0.05 oncological pts vs. non-oncological pts in Student’s *t*-test, **^b^** *p* < 0.05 any time point vs. before alloHSCT.

**Table 3:** The degree of eGFR decrease according to pRIFLE (pediatric Risk, Injury, Failure, Loss and End stage kidney disease) criteria in examined groups in various time points.

Time after AlloHSCT	”Grade zero” (↓ eGFR < 25%) (*n* (%))		Risk (↓ eGFR > 25%) (*n* (%))		Injury (↓ eGFR > 50%) (*n* (%))	
	Onco	Non-onco	Onco	Non-onco	Onco	Non-onco
**1 day**	6 (6.7)	8 (17.8)	1 (1.1)	0	1 (1.1)	1 (2.2)
**1 week**	12 (13.5)	5 (11.1)	0	1 (2.2)	0	0
**2 weeks**	17 (19.1)	12 (26.7)	7 (7.9)	4 (8.9)	1 (1.1)	0
**3 weeks**	29 (32.6)	16 (35.6)	13 (14.6)	3 (6.7)	1 (1.1)	0
**4 weeks**	39 (43.8)	12 (26.7)	16 (18.0)	9 (20.0)	0	0
**8 weeks**	37 (41.6)	14 (31.1)	31 (34.8)	8 (17.8) *	2 (2.2)	1 (2.2)
**3 months**	37 (41.6)	17 (37.8)	21 (23.6)	8 (17.8)	6 (6.7)	1 (2.2)
**6 months**	41 (46.1)	19 (42.2)	18 (20.2)	5 (11.1)	0	0

* *p* < 0.05 oncological vs. non-oncological patients in chi-square Pearson test.

## Data Availability

The datasets generated and analysed during the current study are available from the corresponding author on reasonable request.

## References

[1] Slatter M.A., Gennery A.R. (2008). Hematopoietic cell transplantation in primary immunodeficiency—Conventional and emerging indications. Exp. Rev. Clin. Immunol..

[2] Xu L., Chen H., Chen J., Han M., Huang H., Lai Y., Liu D., Liu Q., Liu T., Jiang M. (2018). The consensus on indications, conditioning regimen, and donor selection of allogeneic hematopoietic cell transplantation for hematological diseases in China—recommendations from the Chinese Society of Hematology. J. Hematol. Oncol..

[3] Sureda A., Bader P., Cesaro S., Dreger P., Duarte R.F., Dufour C., Falkenburg J.H.F., Farge-Bancel D., Gennery A., Kroger N. (2015). Indications for allo- and auto-HSCT for haematological diseases, solid tumours and immune disorders: Cur-rent practice in Europe. Bone Marrow Transplant..

[4] Hołowiecki J. (2008). Indications for hematopoietic stem cell transplantation. Pol. Arch. Intern. Med..

[5] Hierlmeier S., Eyrich M., Wölfl M., Schlegel P.-G., Wiegering V. (2018). Early and late complications following hematopoietic stem cell transplantation in pediatric patients—A retrospective analysis over 11 years. PLoS ONE.

[6] Sahin U., Toprak S.K., Atilla P.A., Atilla E., Demirer T. (2016). An overview of infectious complications after allogeneic hematopoietic stem cell transplantation. J. Infect. Chemother..

[7] Goldstein M.J., Locatelli F., Mielke S., Porter D., Schechter T., Shekhovtsova Z., Ferrara J.L.M., Levine J.E. (2016). International, multi-center standardization of acute graft versus host disease clinical data collection: A report from the MAGIC consortium. Biol. Blood Marrow Transplant..

[8] Çıkı K., Doğru D., Kuşkonmaz B., Emiralioğlu N., Yalçın E., Özçelik U., Uçkan-Çetinkaya D., Kiper N. (2019). Pulmonary complications following hematopoietic stem cell transplantation in children. Turk. J. Pediatr..

[9] Wanchoo R., Stotter B.R., Bayer R.L., Jhaveri K.D. (2019). Acute kidney injury in hematopoietic stem cell transplantation. Curr. Opin. Crit. Care.

[10] Ando M. (2018). An Overview of Kidney Disease Following Hematopoietic Cell Transplantation. Intern. Med..

[11] Ileri T., Ertem M., Ozcakar Z.B., Ince E.U., Biyikli Z., Uysal Z., Ekim M., Yalcinkaya F. (2010). Prospective evaluation of acute and chronic renal function in children following matched related donor hematopoietic stem cell transplantation. Pediatr. Transplant..

[12] Koh K.-N., Sunkara A., Kang G., Sooter A., Mulrooney D.A., Triplett B., Onder A.M., Bissler J., Cunningham L.C. (2018). Acute Kidney Injury in Pediatric Patients Receiving Allogeneic Hematopoietic Cell Transplantation: Incidence, Risk Factors, and Outcomes. Biol. Blood Marrow Transplant..

[13] Raina R., Herrera N., Krishnappa V., Sethi S.K., Deep A., Kao W.-M., Bunchman T., Abu-Arja R. (2017). Hematopoietic stem cell transplantation and acute kidney injury in children: A comprehensive review. Pediatr. Transplant..

[14] Didsbury M.S., Mackie F.E., Kennedy S.E. (2015). A systematic review of acute kidney injury in pediatric allogeneic hematopoietic stem cell recipients. Pediatr. Transplant..

[15] Kizilbash S.J., Kashtan C.E., Chavers B.M., Cao Q., Smith A.R. (2016). Acute Kidney Injury and the Risk of Mortality in Children Undergoing Hematopoietic Stem Cell Transplantation. Biol. Blood Marrow Transplant..

[16] Sutherland S.M., Byrnes J.J., Kothari M., Longhurst C.A., Dutta S., Garcia P., Goldstein S.L. (2015). AKI in Hospitalized Children: Comparing the pRIFLE, AKIN, and KDIGO Definitions. Clin. J. Am. Soc. Nephrol..

[17] Augustynowicz M., Bargenda-Lange A., Kałwak K., Zwolińska D., Musiał K. (2019). Markers of acute kidney injury in children undergoing hematopoietic stem cell transplantation. Adv. Clin. Exp. Med..

[18] Musiał K., Augustynowicz M., Miśkiewicz-Migoń I., Kałwak K., Ussowicz M., Zwolińska D. (2020). Clusterin as a New Marker of Kidney Injury in Children Undergoing Allogeneic Hematopoietic Stem Cell Transplantation—A Pilot Study. J. Clin. Med..

[19] Schwartz G.J., Munoz A., Schneider M.F., Mak R.H., Kaskel F., Warady B.A., Furth S.L. (2009). New equations to estimate GFR in children with CKD. J. Am. Soc. Nephrol..

[20] Caliskan Y., Besisik S.K., Sargin D., Ecder T. (2006). Early renal injury after myeloablative allogeneic and autologous hematopoietic cell transplantation. Bone Marrow Transplant..

[21] Cachat F., Combescure C., Cauderay M.J., Girardin E., Chehade H. (2015). A Systematic Review of Glomerular Hyperfiltration Assessment and Definition in the Medical Literature. Clin. J. Am. Soc. Nephrol..

[22] Iduoriyekemwen N.J., Ibadin M.O., Aikhionbare H.A., Idogun S.E., Abiodun M.T. (2019). Glomerular hyperfiltration in excess weight adolescents. Niger. J. Clin. Pr..

[23] Krishnappa V., Gupta M., Manu G., Kwatra S., Owusu O.-T., Raina R. (2016). Acute Kidney Injury in Hematopoietic Stem Cell Transplantation: A Review. Int. J. Nephrol..

[24] Kanduri S.R., Cheungpasitporn W., Thongprayoon C., Bathini T., Kovvuru K., Garla V., Medaura J., Vaitla P., Kashani K.B. (2020). Incidence and mortality of acute kidney injury in patients undergoing hematopoietic stem cell transplantation: A systematic review and meta-analysis. QJM Int. J. Med..

[25] Filler G., Lee M. (2018). Educational review: Measurement of GFR in special populations. Pediatr. Nephrol..

[26] Zachwieja K., Korohoda P., Krasowska-Kwiecień A., Kwinta-Rybicka J., Miklaszewska M., Moczulska A., Goździk J., Drożdż D. (2019). The variability of glomerular filtration rate (eGFR) in children after hematopoietic stem cell transplantation in one year observation. Przegl. Lek..

[27] Basu R.K., Kaddourah A., Terrell T., Mottes T., Arnold P., Jacobs J., Andringa J., Goldstein S.L. (2015). Assessment of Worldwide Acute Kidney Injury, Renal Angina and Epidemiology in Critically Ill Children (AWARE): Study protocol for a prospective observational study. BMC Nephrol..

[28] Kwatra N.S., Meany H.J., Ghelani S.J., Zahavi D., Pandya N., Majd M. (2016). Glomerular hyperfiltration in children with cancer: Prevalence and a hypothesis. Pediatr. Radiol..

[29] Tiburcio F.R., Rodrigues K.E., Belisario A.R., Simoes-e-Silva A.C. (2018). Glomerular hyperfiltration and beta-2 microglobulin as biomarkers of incipient renal dysfunction in cancer survivors. Future Sci. OA.

[30] Wei J., Zhang J., Jiang S., Wang L., Persson A.E.G., Liu R. (2019). High protein diet-induced glomerular hyperfiltration id de-pendent on NOS1beta in the macula densa via tubuloglomerular feedback response. Hypertension.

[31] Vallon V., Thomson S.C. (2020). The tubular hypothesis of nephron filtration and diabetic kidney disease. Nat. Rev. Nephrol..

